# Supplementary figures and images for: Genotype imputation using the Positional Burrows Wheeler Transform
Source: PLoS Genet. 2020 Nov 16;16(11):e1009049. doi: 10.1371/journal.pgen.1009049 (PMC7704051; doi:10.1371/journal.pgen.1009049)

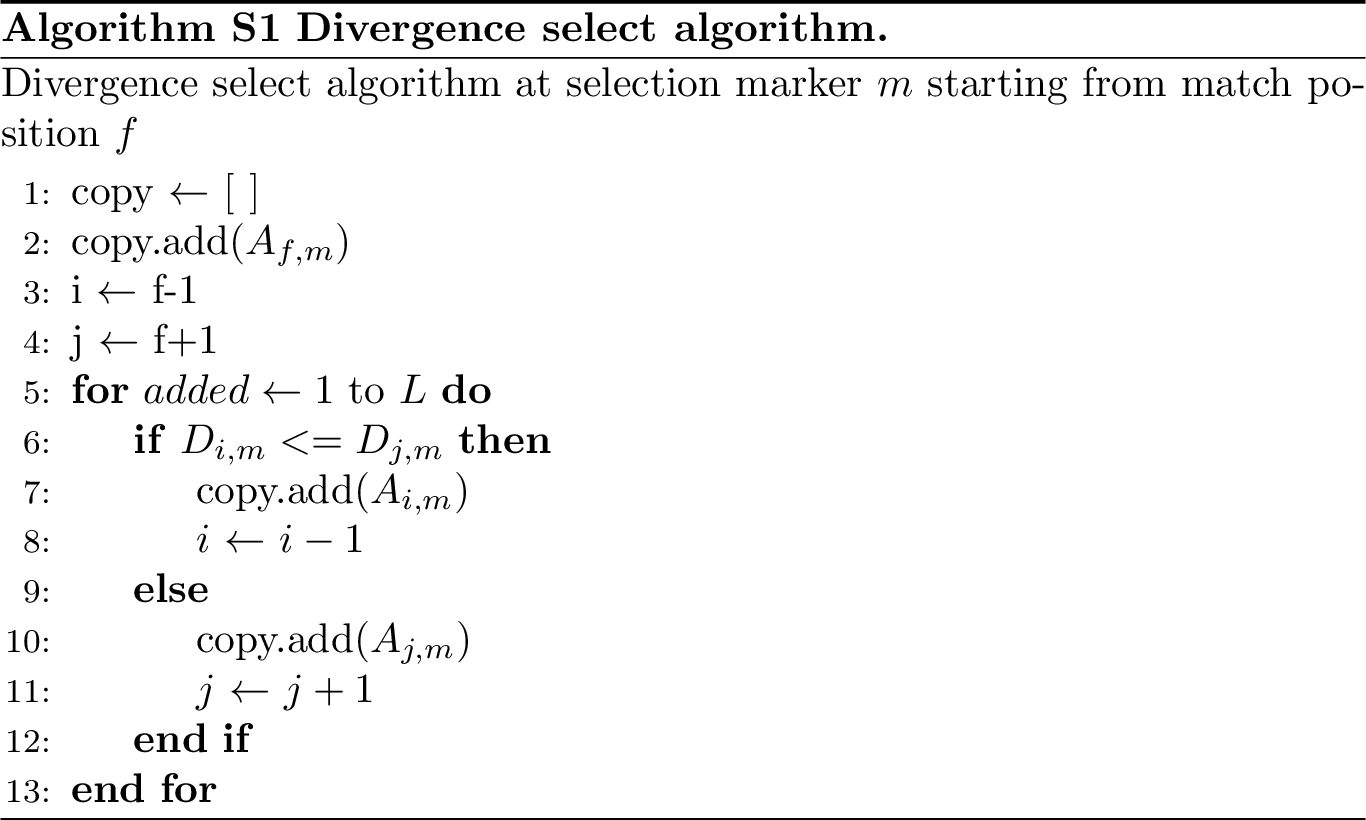

Supplement: S1 Algorithm — (TIF) [file pgen.1009049.s001.tif]

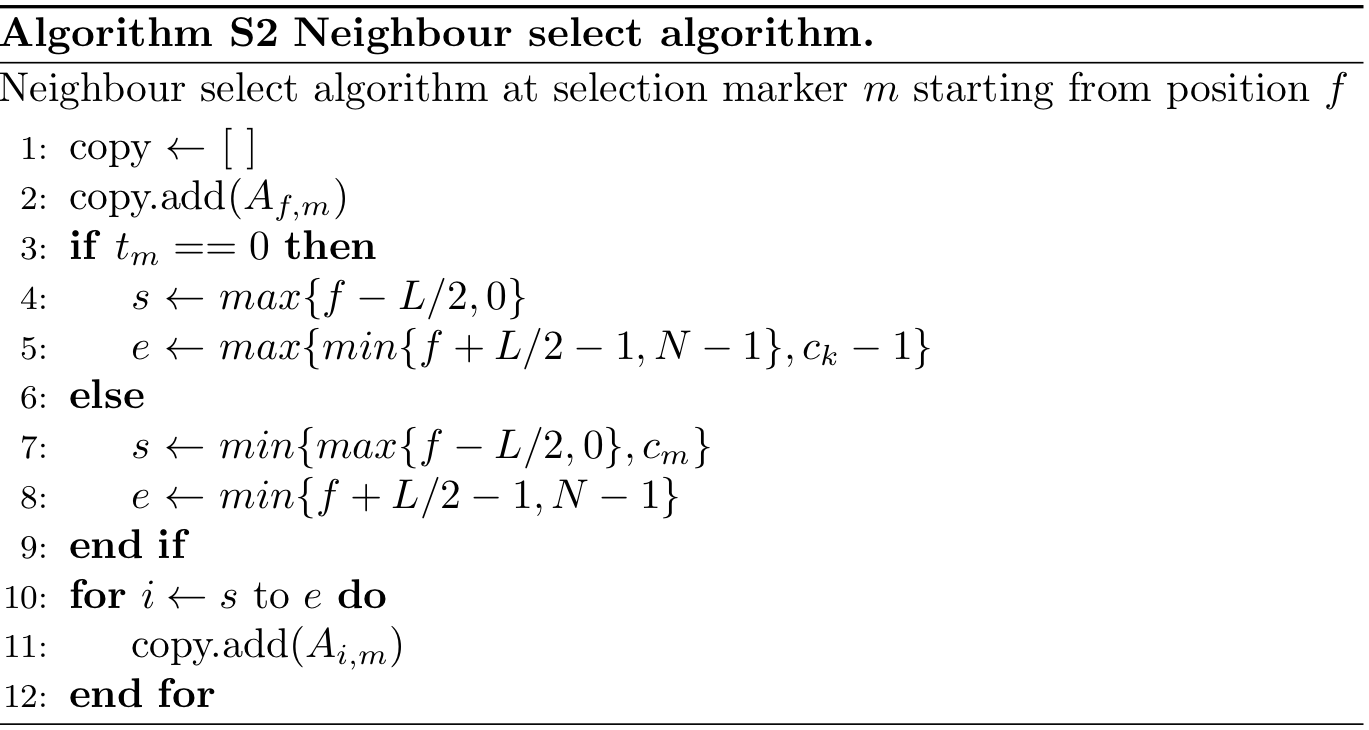

Supplement: S2 Algorithm — (TIF) [file pgen.1009049.s002.tif]

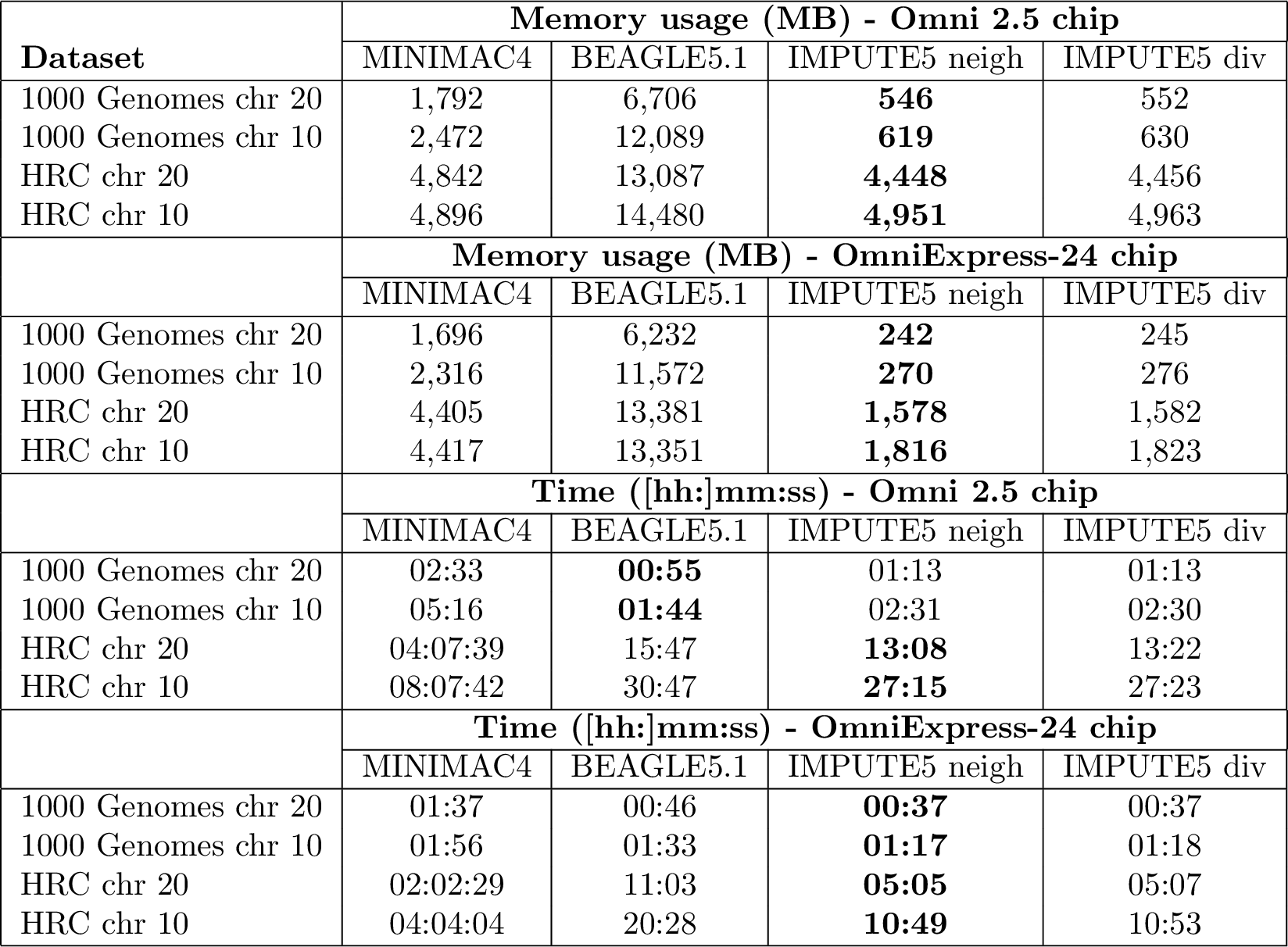

Supplement: S1 Table — Memory usage and total time to impute a whole chromosome (chr 10 and chr 20) for 52 target samples when using the 1000 Genomes reference panel and 1,000 target samples when using the HRC reference panel. MINIMAC4 was run on chunks of size 20 Mb while BEAGLE5 and IMPUTE5 on chunks of size 20 cM. Time is shown using the format mm:ss. Bold font is used to indicate the method with the lowest time. (TIF) [file pgen.1009049.s003.tif]

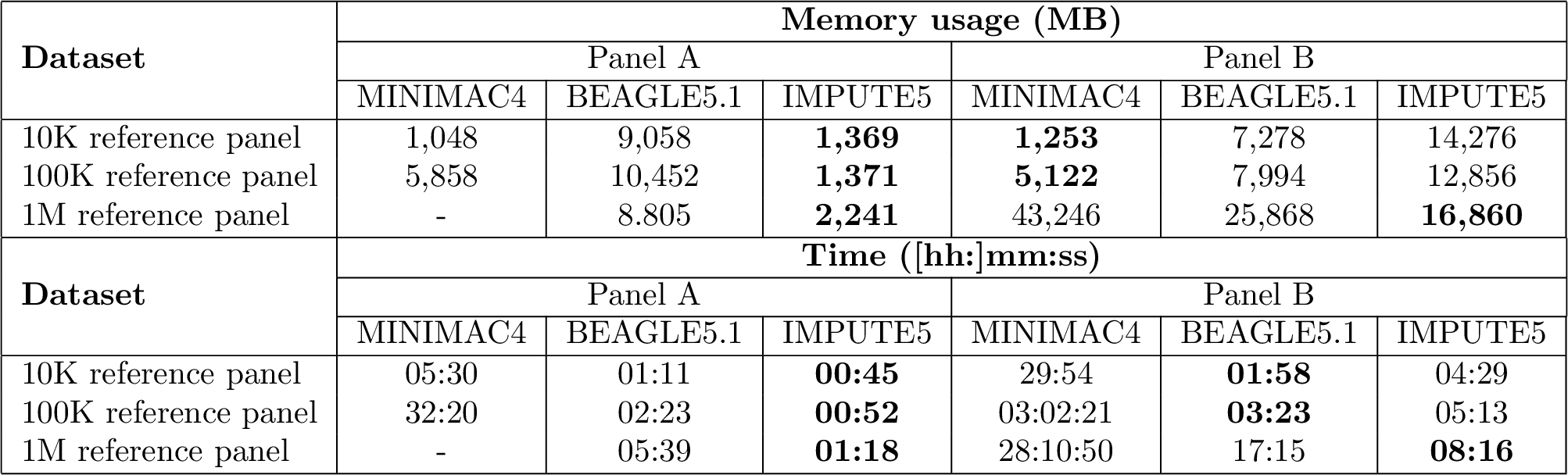

Supplement: S2 Table — Total time to impute 1, 000 target samples in a 10Mb window using simulation data in Panel A and Panel B dataset. Time is shown using the format mm:ss. Bold font is used to indicate the method with the lowest time. Minimac4 was not able to run using the Panel A 1M reference panel due to time constraints in the construction of the m3vcf file. (TIF) [file pgen.1009049.s004.tif]

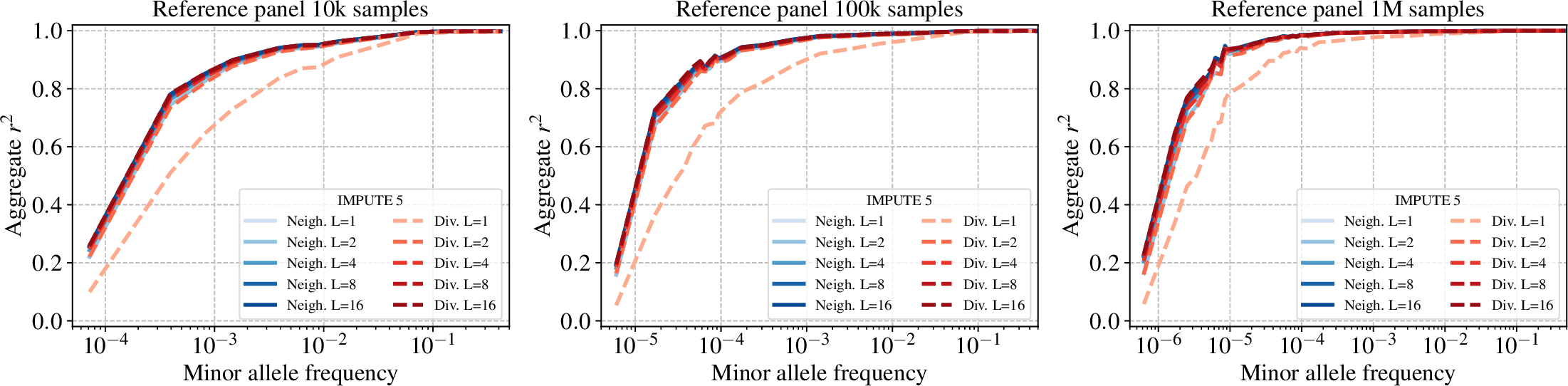

Supplement: S1 Fig — Imputation accuracy when imputing genotypes from a simulated reference panel of 10K, 100K and 1M UK-European reference samples (Panel A). The horizontal axis in each panel is on a log scale. (TIF) [file pgen.1009049.s005.tif]

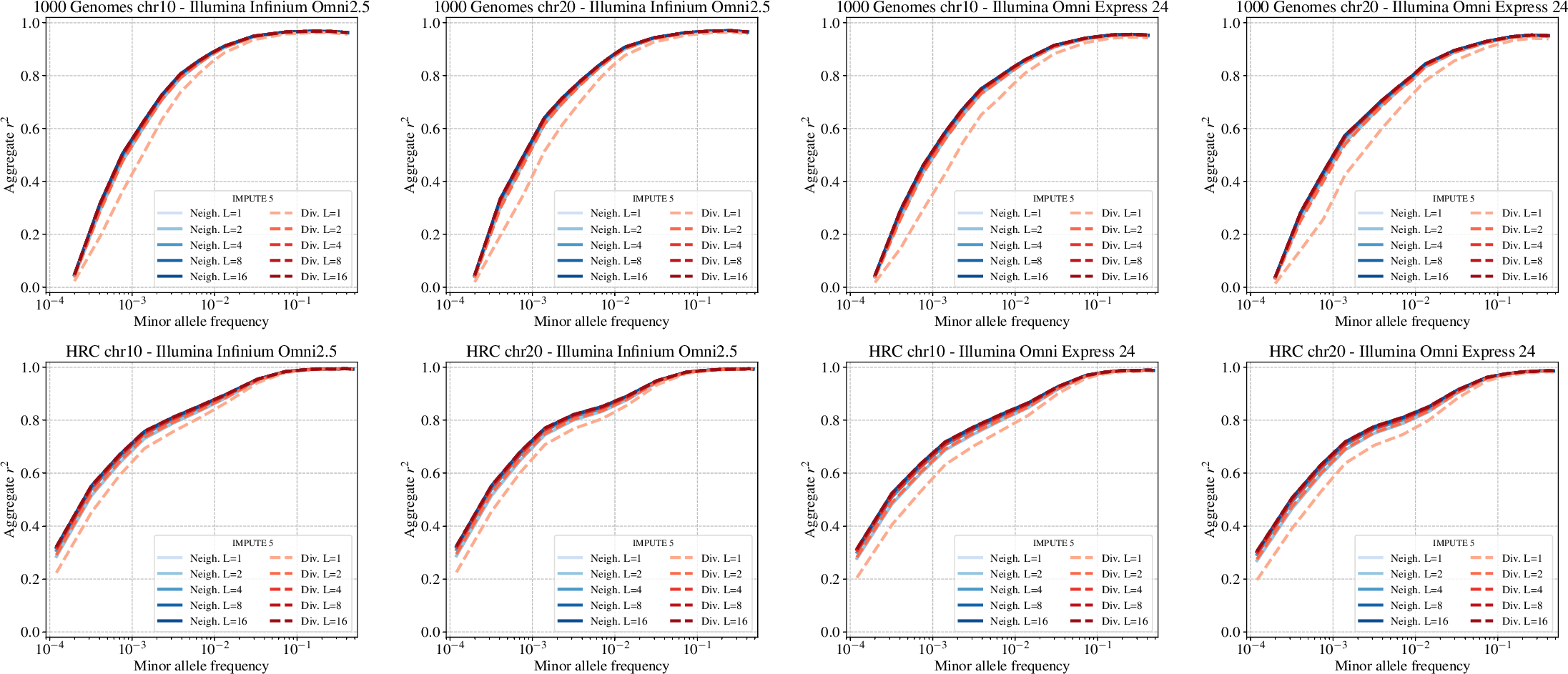

Supplement: S2 Fig — Genotype imputation accuracy when imputing genotypes using the 1000 Genomes Project reference panel (n = 2452) and the Haplotype Reference Consortium reference panel (n = 31470). The horizontal axis in each panel is on a log scale. (TIF) [file pgen.1009049.s006.tif]

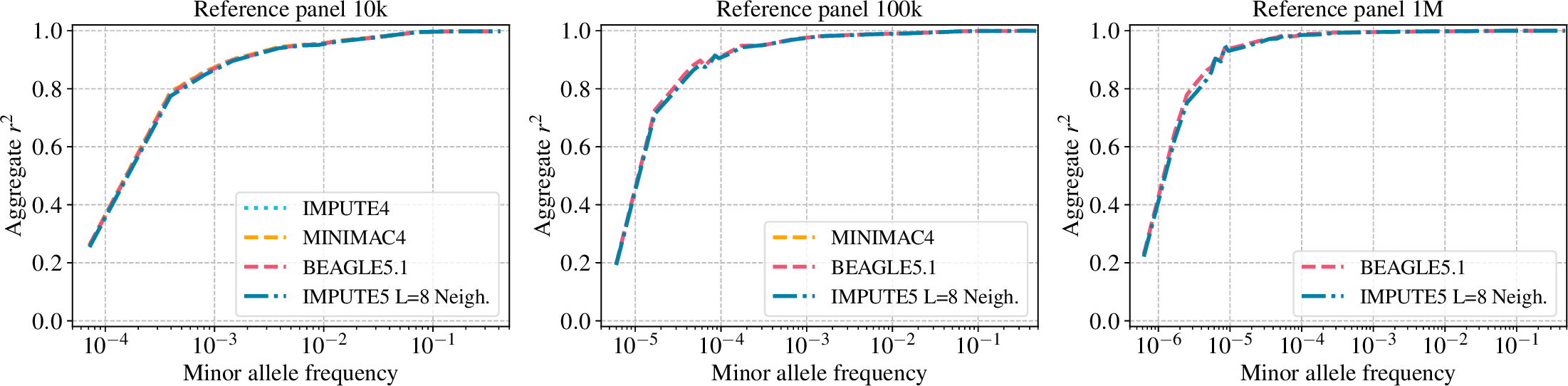

Supplement: S3 Fig — Genotype imputation accuracy when imputing genotypes using the 1000 Genomes Project reference panel (n = 2452) and the Haplotype Reference Consortium reference panel (n = 31470) for diffent values of the parameter L using the neighbour selection algorithm and the divergence selection algorithm. The horizontal axis in each panel is on a log scale. (TIF) [file pgen.1009049.s007.tif]

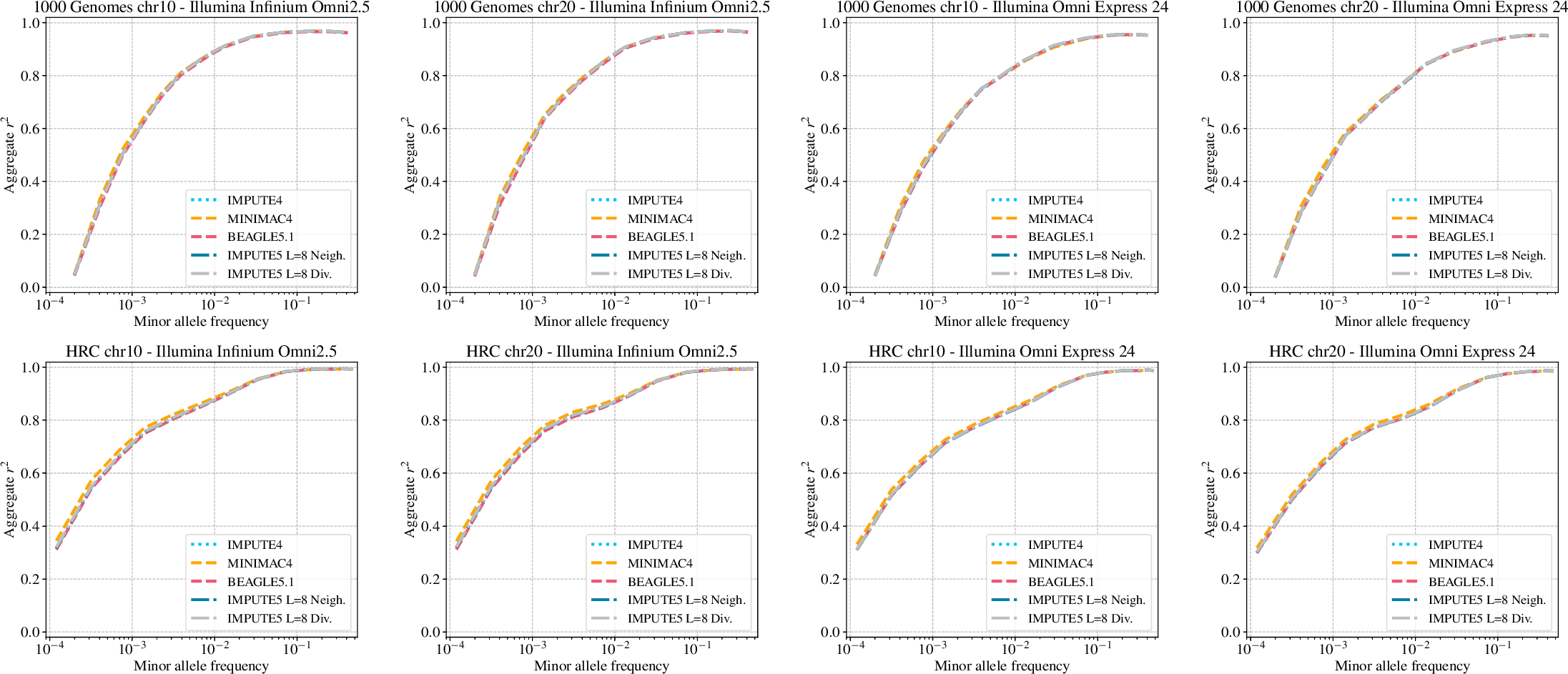

Supplement: S4 Fig — Imputation accuracy when imputing genotypes using the 1000 Genomes Project reference panel (n = 2452) and the Haplotype Reference Consortium reference panel (n = 31470) for different values of the parameter L using the neighbour selection algorithm and the divergence selection algorithm. The horizontal axis in each panel is on a log scale. (TIF) [file pgen.1009049.s008.tif]

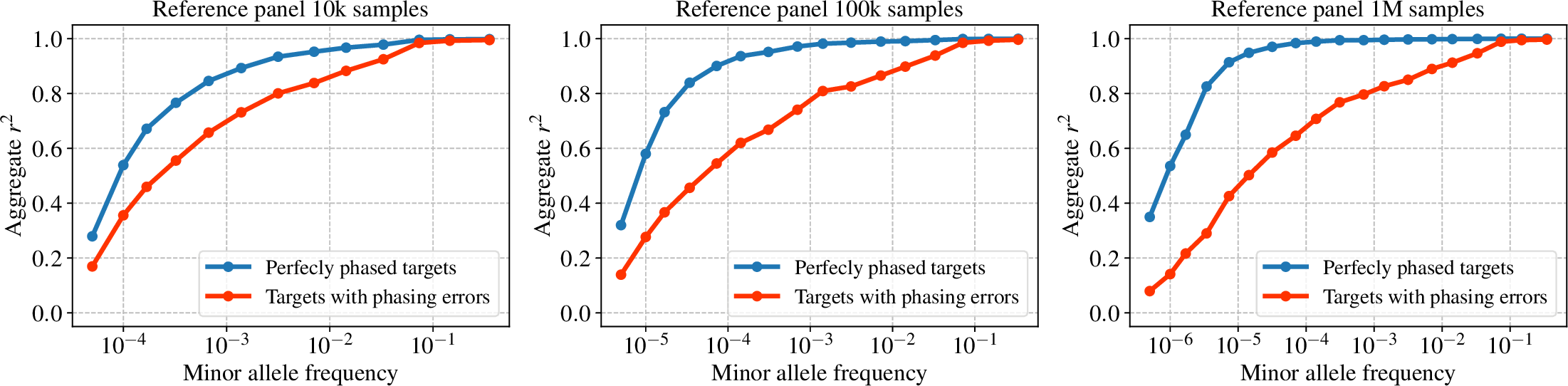

Supplement: S5 Fig — Imputation accuracy when imputing 1000 target samples from a simulated reference panel of 10K, 100K and 1M UK-European samples (Panel A) with no phasing errors (blue) and with a ≈ 2% switch error rate (red). The horizontal axis in each panel is on a log scale. (TIF) [file pgen.1009049.s009.tif]

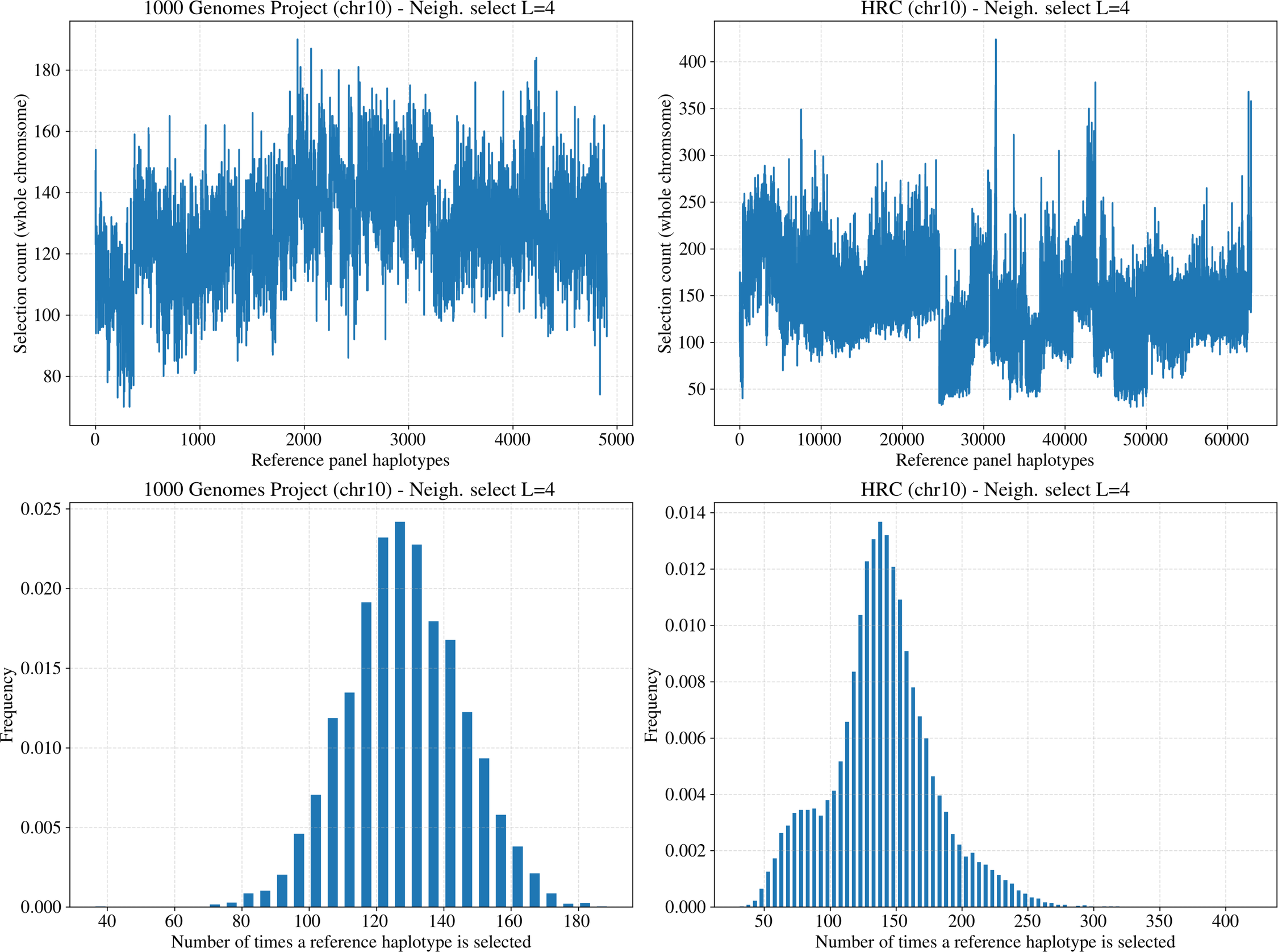

Supplement: S6 Fig — Count of the number each reference haplotypes selected along the ten imputation chunks of chromosome 10 for the 1000 Genomes Project and HRC reference panel (top). Histogram of the selected counts for the two datasets (bottom). (TIF) [file pgen.1009049.s010.tif]

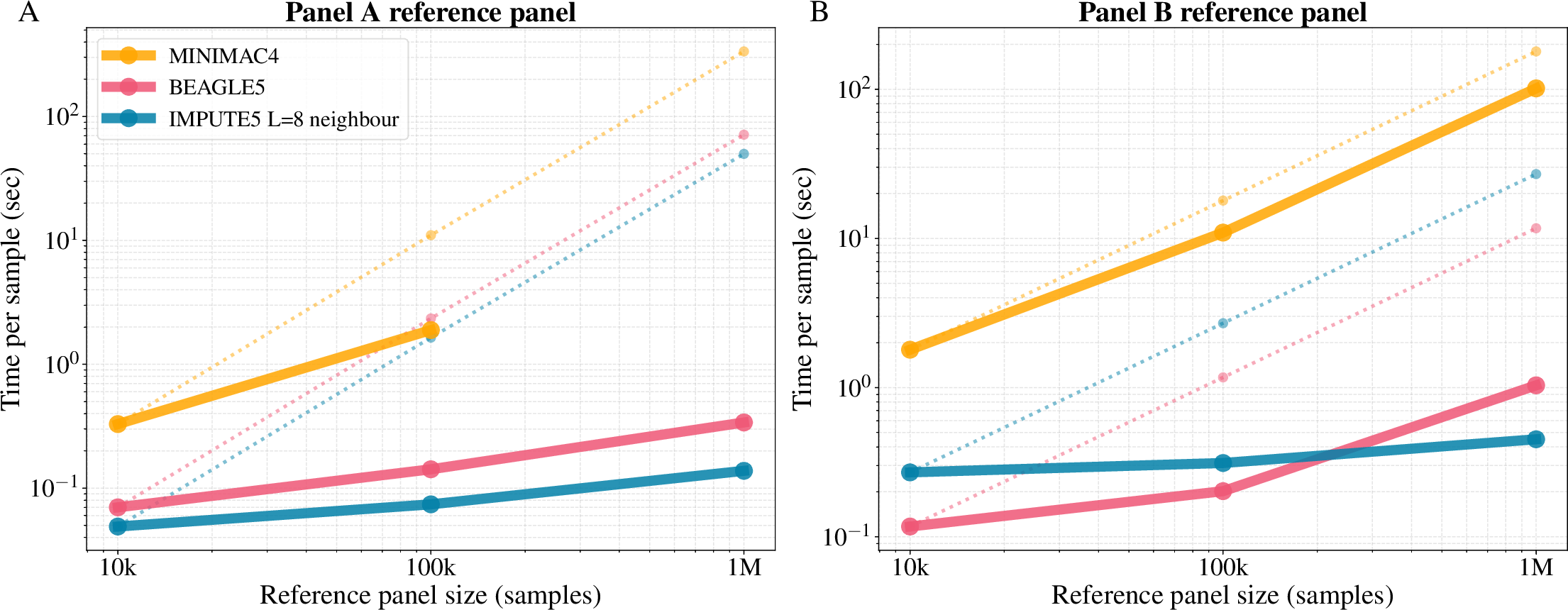

Supplement: S7 Fig — Per-sample CPU time when imputing a 10 Mb region from 10K, 100K and 1M simulated UK-European reference samples into 1,000 target samples using one computational thread. (A) Imputation time when using Panel A dataset (3,333 target markers). (B) Imputation time when using Panel B dataset (33,333 target markers). Axes are on log scale. Hypothetical linear scaling of MINIMAC4, BEAGLE5 and IMPUTE5 are shown as dotted lines, generated by projecting the time using the 10K reference panel. Minimac4 was not able to run using the Panel A 1M reference panel due to time constraints in the construction of the m3vcf file. (TIF) [file pgen.1009049.s011.tif]

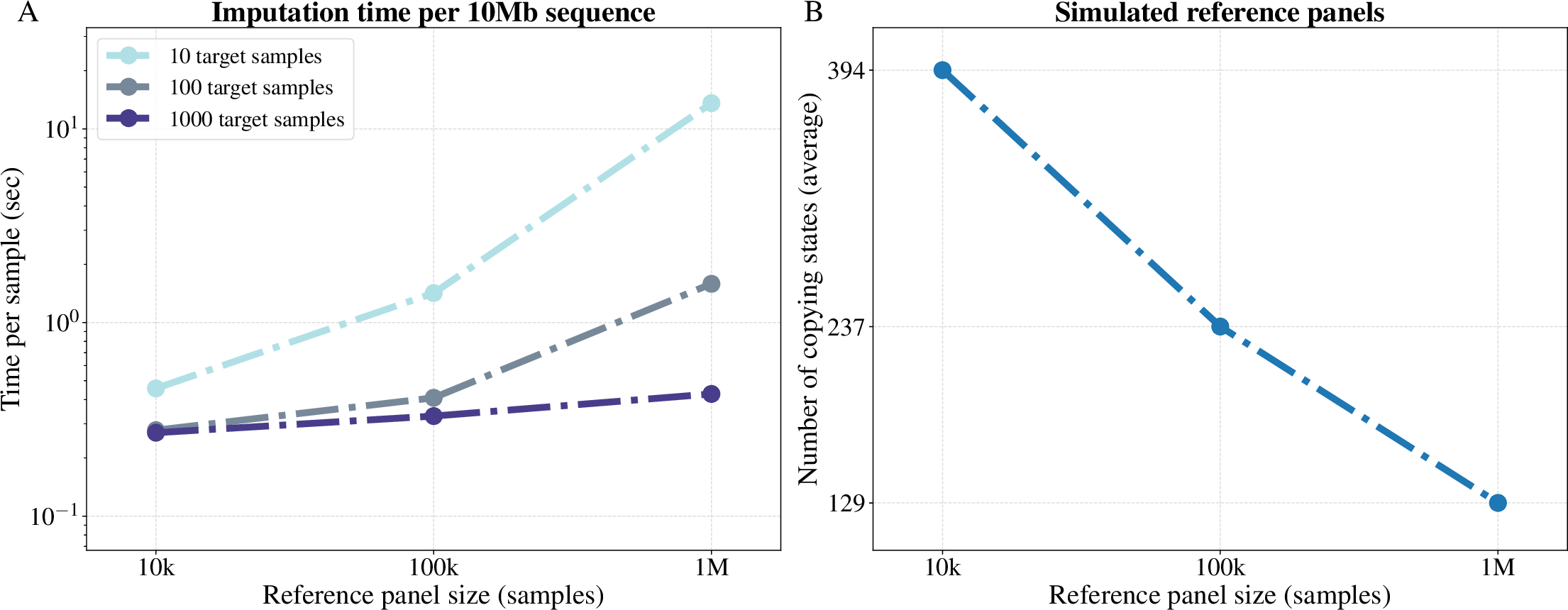

Supplement: S8 Fig — (A) Time per sample spent to impute a marker in a 10Mb region for reference panel size 10K, 100K, 1000K, when imputing 10, 100 and 1000 target samples. The vertical axis is on a log scale. (B) Mean number of copying states selected for the simulated reference panels. The number of selected states decreases by increasing the size of the reference panel, showing sub-linear scaling. Time and number of conditioning states are obtained with neighbours select and L = 8. (TIF) [file pgen.1009049.s012.tif]
